# Asymptomatic and submicroscopic *Plasmodium* infections in an area before and during integrated vector control in Homa Bay, western Kenya

**DOI:** 10.1186/s12936-022-04288-2

**Published:** 2022-09-24

**Authors:** Collince J. Omondi, Wilfred O. Otambo, David Odongo, Kevin O. Ochwedo, Antony Otieno, Shirley A. Onyango, Pauline Orondo, Benyl M. Ondeto, Ming-Chieh Lee, Daibin Zhong, James W. Kazura, Andrew K. Githeko, Guiyun Yan

**Affiliations:** 1 Department of Biology, Faculty of Science and Technology, University of Nairobi, Nairobi, Kenya; 2 Sub-Saharan International Center of Excellence for Malaria Research, Homa Bay, Kenya; 3 Department of Zoology, Maseno University, Kisumu, Kenya; 4 Department of Zoological Sciences, School of Science and Technology, Kenyatta University, Nairobi, Kenya; 5 Center for Global Health and Diseases, Case Western Reserve University, Cleveland, OH 44106 USA; 6 Climate and Human Health Research Unit, Center for Global Health Research, Kenya Medical Research Institute, Kisumu, Kenya; 7 Program in Public Health, College of Health Sciences, University of California, Irvine, CA 92697 USA

**Keywords:** *Plasmodium falciparum*, Asymptomatic malaria, Submicroscopic infection, Vector control, Kenya

## Abstract

**Background:**

Long-lasting insecticidal nets (LLINs) have been the primary vector control strategy until indoor residual spraying (IRS) was added in Homa Bay and Migori Counties in western Kenya. The objective of this study was to evaluate the impact of LLINs integrated with IRS on the prevalence of asymptomatic and submicroscopic *Plasmodium* infections in Homa Bay County.

**Methods:**

A two-stage cluster sampling procedure was employed to enroll study participants aged ≥ 6 months old. Four consecutive community cross-sectional surveys for *Plasmodium* infection were conducted in residents of Homa Bay county, Kenya. Prior to the start of the study, all study households received LLINs, which were distributed between June 2017 and March 2018. The first (February 2018) and second (June 2018) surveys were conducted before and after the first round of IRS (Feb–Mar 2018), while the third (February 2019) and fourth (June 2019) surveys were conducted before and after the second application of IRS (February–March 2019). Finger-prick blood samples were obtained to prepare thick and thin smears for microscopic determination and qPCR diagnosis of *Plasmodium* genus.

**Results:**

*Plasmodium* spp. infection prevalence by microscopy was 18.5% (113/610) before IRS, 14.2% (105/737) and 3.3% (24/720) after the first round of IRS and 1.3% (11/849) after the second round of IRS (p < 0.0001). Submicroscopic (blood smear negative, qPCR positive) parasitaemia reduced from 18.9% (115/610) before IRS to 5.4% (46/849) after IRS (p < 0.0001). However, the proportion of PCR positive infections that were submicroscopic increased from 50.4% (115/228) to 80.7% (46/57) over the study period (p < 0.0001). Similarly, while the absolute number and proportions of microscopy positives which were asymptomatic decreased from 12% (73/610) to 1.2% (9/849) (p < 0.0001), the relative proportion increased. Geometric mean density of *P. falciparum* parasitaemia decreased over the 2-year study period (p < 0.0001).

**Conclusions:**

These data suggest that two annual rounds of IRS integrated with LLINs significantly reduced the prevalence of *Plasmodium* parasitaemia, while the proportion of asymptomatic and submicroscopic infections increased. To reduce cryptic *P. falciparum* transmission and improve malaria control, strategies aimed at reducing the number of asymptomatic and submicroscopic infections should be considered.

## Background

Despite increased malaria control interventions, malaria morbidity and mortality remain high, making the disease a major public health concern in sub-Saharan Africa [1–3]. Out of the 229 million malaria cases in 2019, 94% of them were from sub-Saharan Africa. In Kenya, malaria remains a major public health challenge, with approximately 70% of the population at risk, resulting in about 13–15% of outpatient consultations [4]. Malaria transmission is perennial with parasite prevalence consistently above 20% in the Lake Victoria Basin region [5–7]. Morbidity and mortality mostly in young children and pregnant women remains prevalent along the lake region [5, 6]. *Plasmodium falciparum* is the most abundant species (92%) accompanied by *Plasmodium malariae* (6%) and *Plasmodium ovale* at 2% [8].

The fight against malaria has been scaled up along the Lake Victoria Basin region, where the infection rates remain high. For instance, mass distribution of LLINs occurs every 3 years [9]. Besides, indoor residual spraying (IRS) was initiated in 2008 [10], but due to reports of emerging resistance to pyrethroid insecticides by malaria vectors, IRS was suspended in endemic parts of Kenya in 2012 [5, 6, 11]. Since then, Kenya has relied on long-lasting insecticidal nets as the primary vector control until February 2018 when IRS was re-introduced using Actellic 300 CS, an organophosphate insecticide. These control strategies toward malaria in Kenya have been intensified to minimize malaria burden in most affected regions. However, this may be undermined by the residual proportions of both asymptomatic and submicroscopic *Plasmodium* infections in low transmission areas [12, 13]. These infections act as major reservoirs of malaria parasites, thus will sustain transmission. Therefore, formulation of sustainable control strategies to mitigate challenges fueling transmission is very critical and should be regarded as a priority investment.

While the impact of long-lasting insecticidal nets on malaria has been well documented in western Kenya, little is known on the impacts of the addition of IRS on parasite profiles at the population level. Therefore, a study targeting the entire community to identify the prevalence of malaria parasites, asymptomatic and submicroscopic infections was undertaken before the application of indoor residual spraying (IRS, Actellic 300 CS) and during IRS intervention period. Highly sensitive molecular tools were used to detect submicroscopic parasites and improve species identification. Results of this study will guide policy makers in the health, and other concerned stakeholders with regard to enhanced malaria control and management.

## Methods

### Study site

**Fig. 1:**
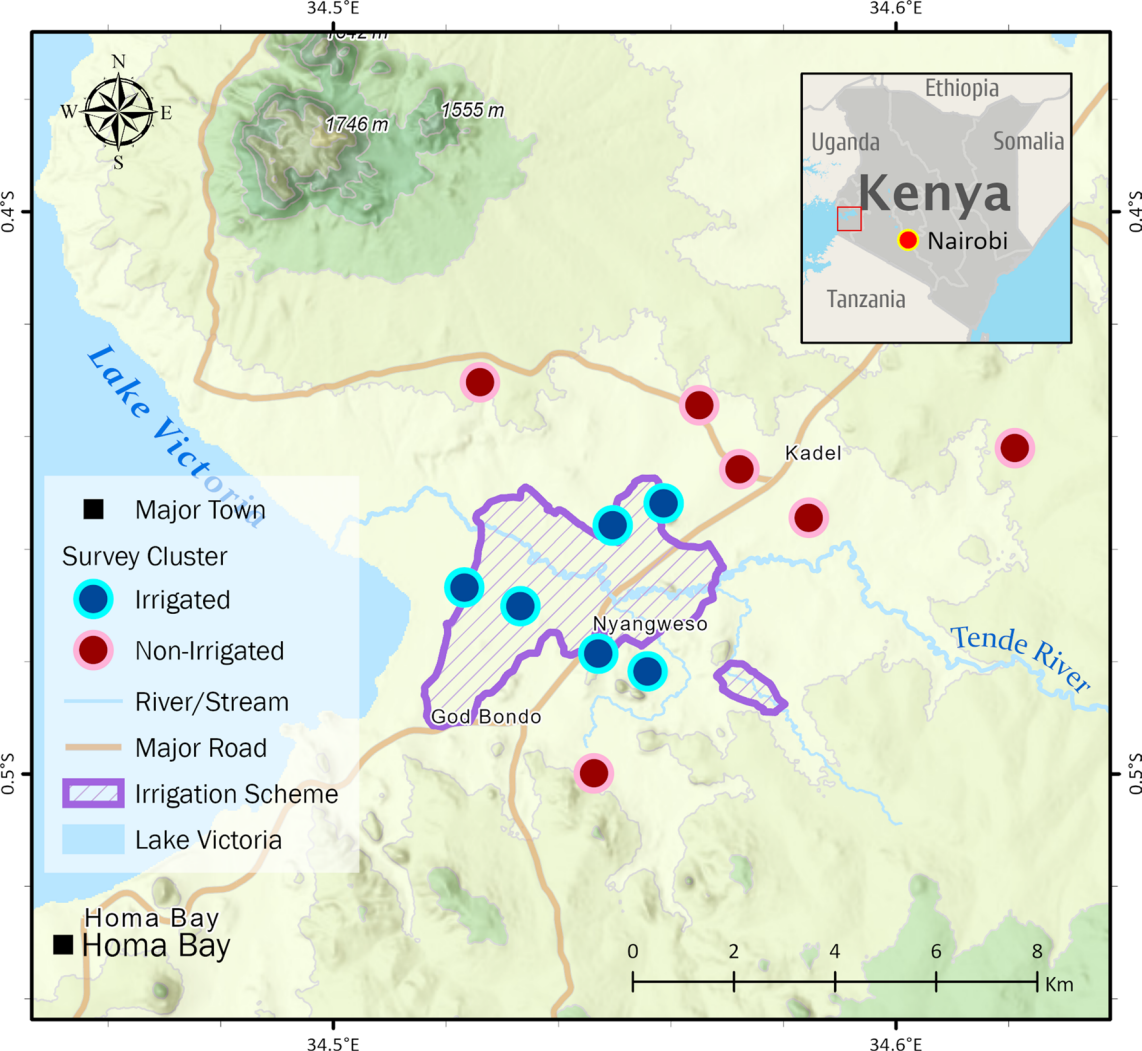
Map of study area showing part of Rangwe and Rachuonyo South sub-counties in Homa Bay county shaded red in the map of Kenya

The study was undertaken in Rachuonyo South and Rangwe sub-counties of Homa Bay County, Kenya located at 0^o^15′–0^o^52′ South latitude and 34°–35° east longitude. The area has two rainy seasons: “long rains” (April–July) and “short rains” (September–November). Annual rainfall is 250 to 1000 mm. The study area is comprised of irrigated and non-irrigated settings. In irrigated area, a concrete channel based multi-crop irrigation scheme was constructed in 2012 to boost subsistence farming. Non-irrigated areas are located 5–10 km away from irrigated areas as indicated in Fig. 1. Between June 2017 and March 2018, the Kenyan Ministry of Health distributed long-lasting insecticidal nets (LLINs) to all malaria endemic and epidemic areas [9]. Following the mass distribution campaign, approximate 85% of all households in the Lake Victoria Basin region owned at least one LLINs [9]. In addition to LLINs, the Ministry implemented annual IRS with the organophosphate pirimiphos methyl 300 CS for concerted malaria vector control in February–March 2018, 2019 and 2020 [10, 14]. Before the introduction of IRS, the Kenyan Ministry of Health estimated the annual *P. falciparum* infection prevalence was 27% [6].

### Sample size determination

The formula proposed by Naing et al. [15] was used to determine sample size. Briefly, assuming malaria prevalence (p) of 27% [6], precision (d) of 0.05 and level of confidence (z) as 1.96, the minimum number of study participants needed was 303. This was arrived at as follows:

$$N = \frac{{Z^{2} P\left( {1 - P} \right)}}{{d^{2} }} \to N = \frac{{1.96^{2} \times 0.27\left( {0.73} \right)}}{{0.05^{2} }}\, = 303 \to 303 \times 2\,\left( {{\text{design}}\,{\text{effect}}} \right) = 606.$$

Due to the cluster sampling procedure, the sample size was multiplied by a design effect as proposed by Cochran [16]. The design effect in most cluster surveys has been reported to be 2 [17] Hence, the minimum number of study participants required was 606. To prevent survey attrition, we decided to recruit 4 participants per family because the average number per household was 4.4, resulting in 80 individuals per cluster and 960 for the entire study region. However, only 610, 737, 720, and 849 participants were tested during the first, second, third and fourth surveys, respectively, due to participants’ unavailability during surveys.

### Cross-sectional surveys

Four community cross-sectional surveys were conducted from February 2018 to June 2019. The first survey was in February 2018 before the first IRS, and the second in June 2018. A similar schedule was executed in 2019 before and after the second IRS. Selection for study participation was based on a two-stage procedure similar to that described by Galway et al. [18]. The first stage involved identification of clusters (essentially villages) as primary sampling units. For example, with the help of chiefs and village heads the study team identified the total number of clusters (30). Based on the number of participants required, 12 clusters were randomly selected from the previously identified 30 clusters. In the second stage, the total number of households in each cluster was determined and assigned unique identification numbers. Twenty households from each cluster were then randomly selected provided the household head accepted to participate. The study team recruited 80 permanent dwellers from each cluster. In every household, 4 participants, aged 6 months and above, who were permanent dwellers and who were willing to participate and sign the informed consent or assent forms (for participants < 18 years), were recruited. In case there were more than 4 eligible participants in a household, the 4 participants were randomly selected. However, in households with less than 4 eligible participants, the next household was selected. A questionnaire was used to collect information such as gender, age and body temperature. An infrared thermometer was aimed at the forehead to capture the body temperature. Individuals who had a fever in the previous 48 h were also recorded in the questionnaire. During the survey period, any study participant who had a fever or tested positive for *Plasmodium* infection was referred to the nearest health center for further examination and treatment.

### Sample collection and microscopic examination

Finger-prick blood samples were obtained from study participants to screen for *Plasmodium* infections by microscopy. Briefly, thick and thin blood smears were stained with 10% Giemsa for 15 min and examined using a microscope. A total of 200 microscopic fields containing leukocytes in the thick smear were examined before declaring a slide negative. For quality control, each slide was examined by two independent microscopists, In the case of discrepancy, a third reader was involved. To estimate the *P. falciparum* density, the number of parasites observed in microscopic fields containing based on the 200 leucocytes on a thick smear. This number was then multiplied by 40 assuming 8000 leukocytes/µL blood [19]. The parasite density was further classified as either low (< 1000 parasites/µL), moderate (1000–4999 parasites/µL), high (5000–99,999 parasites/µL) or hyperparasitaemia (≥ 100,000 parasites/µL) [20].Gametocyte density was also estimated by counting gametocytes against 500 leukocytes in thick smear [21]. This was then converted to gametocytes/µl of blood by multiplying with the standard count of 8000 white blood cells/µl.

### DNA extraction and qPCR detection of parasite infections

Dry blood spots were collected from study participants as described (Wampfler et al. [22]). Briefly, 50 µL of finger-prick blood was spotted on Whatman^®^ 3MM filter paper (3 spots). Filter paper was air dried and kept at 4 °C in sealed plastic bags with a desiccant. DNA was extracted from each filter paper using Chelex resin (chelex -100) as described previously [23] with minor modifications [24]. The study focused on examination of the three *Plasmodium* species (*P. falciparum*, *P. malariae*, and *P. ovale*) since they had been previously reported as the most possible species to exist in study area [8]. Malaria parasite detection was performed on all extracted DNA (all blood smear positives and negatives) using multiplex real-time PCR (qPCR) targeting 18S rRNA gene as described elsewhere [25, 26] with modifications. Briefly, the probes and primers of three species (*P. falciparum*, *P. malariae*, and *P. ovale*) were used for multiplex qPCR. The qPCR was run on the Applied Biosystems QuantStudio 3 Real-Time PCR System (Thermo Fisher Scientific Inc. USA) in a final volume containing 6 µl of PerfeCTa^®^ qPCR ToughMix™, Low ROX™ Master mix (2X), 0.4 µl of the species specific forward and reverse primers (10 µM), 0.5 µl of the species-specific probe, 0.1 µl of double-distilled water and 2 µl of sample DNA. The thermo profile was set as follows; 50 °C for 2 min, (95 °C for 2 min, 95 °C for 3 s and 58 °C for 30 s) for 45 cycles.

### Data analysis

An asymptomatic infection was defined as a *Plasmodium* positive test result with the body temperature below 37.5 °C during the time of blood sample collection or in the last 48 h. Blood samples testing negative by microscopy but positive by qPCR were considered to be submicroscopic. All data collected was entered in Excel spreadsheet, cleaned and malaria prevalence from the study area analysed using GraphPad Prism version 8. Differences in prevalence among 4 cross-sectional surveys and age sets were determined using inferential statistics (Pearson *χ*^2^). Parasite densities were logarithm-transformed (log (x + 1) and mean difference among age groups or survey periods determined using analysis of variance (ANOVA).

## Results

### Demographic characteristics of participants

**Fig. 2:**
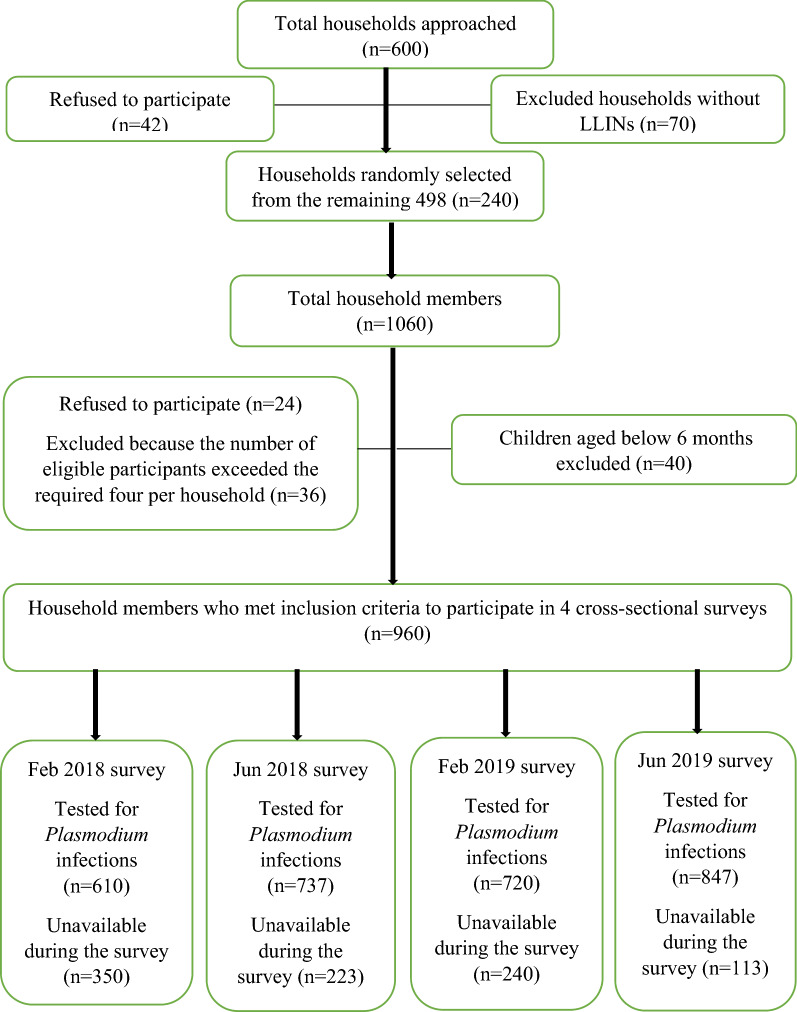
Trial profile diagram showing the study participants recruitment criteria

The study team approached 600 households in the study area during the recruitment stage. However, 42 household heads refused to participate, and 70 household stated that they did not have LLINs and were, therefore, excluded. A total of 240 households were chosen at random from the remaining 498 households to participate in the study. These 240 households had a total population of 1060 people. However, 24 of them refused to participate, 40 children under the age of 6 months were excluded, and 36 people from households with more than 4 eligible participants were also excluded. Finally, the total number of participants who met the inclusion criteria was 960 as indicated in Fig. 2. Due to unavailability of participants during surveys, the participants who were tested for *Plasmodium* species infections during the first, second, third, and fourth survey, were 610, 737, 720, and 849 study participants respectively. Age group distribution during the survey period are shown in Table 1.

**Table 1:** Demographic characteristics of the study participants, parasite density, asymptomatic and submicroscopic *Plasmodium* infections during the 4 cross-sectional surveys

Variable	Survey period			
	Feb 2018 (dry season)	June 2018 (wet season)	Feb 2019 (dry season)	June 2019 (wet season)
Age group (yrs.) n (%)				
< 5	96 (15.7)	139 (18.9)	163 (22.6)	144 (17.0)
5–14	210 (34.4)	285 (38.7)	250 (34.7)	260 (30.6)
≥ 15	304 (49.8)	313 (42.5)	307 (42.6)	445 (52.4)
Microscopic malaria parasite prevalence % (n)	18.5 (113/610)	14.2 (105/737)	3.3 (24/720)	1.3 (11/849)
Malaria parasite prevalence by qPCR % (n)	37.4 (228/610)	25.1 (185/737)	10.4 (75/720)	6.7 (57/849)
Geometric mean parasite density (parasites/µl of blood)	2229	353	578	184
*Plasmodium falciparum* parasite density category % (N)	100% (113)	100% (102)	100% (24)	100% (11)
Low % (n)	33.6 (38)	77.8 (77)	58.3 (14)	90.9 (10)
Moderate % (n)	36.3 (41)	19.2 (19)	41.7 (10)	9.1 (1)
High % (n)	27.4 (31)	3.0 (3)	0.0 (0)	0.0 (0)
Hyper % (n)	2.7 (3)	(0)	0.0 (0)	0.0 (0)

*Low*  < 1000 parasites/µL, *moderate* 1000–4999 parasites/µL, *high* 5000–99,999 parasites/µL, *hyperparasitaemia* ≥ 100,000 parasites/µL

### Parasite prevalence

During the first survey (dry season), conducted in February 2018, the parasite prevalence by microscopy and qPCR was 18.5% (113/610) and 37.4% (228/610), respectively. A significant reduction in prevalence was recorded during the second cross-sectional survey (wet season) by both microscopy and qPCR. For instance, parasite prevalence reduced to 14.2% (105/737) (*χ*^2^ = 5.117, df = 1, p = 0.02) by microscopy and 25.1% (185/737) (*χ*^2^ = 23.08, df = 1, p < 0.0001) by qPCR. Further decline in parasite prevalence was reported in the third survey with microscopy and qPCR recording 3.3% (24/720) and 10.4% (75/720) respectively. During the last survey, malaria parasite prevalence was finally reduced to 1.3% (11/849) and 6.7% (57/849) by microscopy and qPCR respectively. Overall, the reduction in parasite prevalence during the 4 cross-sectional surveys was significant by microscopy (*χ*^2^ = 186.9, df = 3, p < 0.0001) and by qPCR (*χ*^2^ = 266.2, df = 3, p < 0.0001) as indicated in Table 1.

**Fig. 3:**
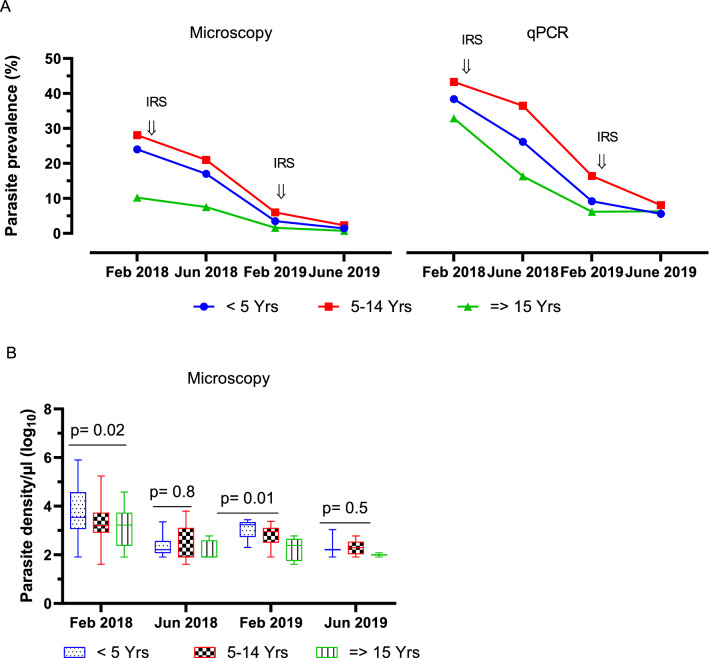
Graph 2A shows the prevalence of *Plasmodium* infection by microscopy and qPCR among age groups during the 4 surveys. Graph 2B indicates the parasite density among age groups by microscopy

Parasite prevalence as determined by both microscopy and qPCR, varied significantly among age groups during the first, second and third survey. For instance, during the first survey, *Plasmodium* infection rate among participants aged < 5, 5–14 and ≥ 15 years old by microscopy was 24% (23/96), 28.1% (59/210), and 10.2% (31/304), respectively (*χ*^2^ = 28.6, df = 2, p < 0.0001). During the second survey, parasite prevalence among children aged below 5, 5–14 and those aged ≥ 15 years was 15.8% (22/139), 20.7% (59/285), and 7.7% (24/313), respectively (*χ*^2^ = 25.1, df = 2, p < 0.0001). Similarly, during the third survey, *Plasmodium* infection rate among < 5, 5–14, and ≥ 15 years old was 2.4% (4/163), 6% (15/250) and 1.6% (5/307), respectively (*χ*^2^ = 8.6, df = 2, p = 0.01). However, during the fourth survey, the parasite prevalence among < 5, 5–14, and ≥ 15 years old was 1.4% (2/144), 2.3% (6/260), and 0.7% (3/445), respectively (*χ*^2^ = 1.9, df = 2, p = 0.39) (Fig. 3). A similar pattern of variation in parasite prevalence among age groups was recorded by qPCR during the 4 cross-sectional surveys as shown in Fig. 3A.

### *Plasmodium falciparum* parasite density and category

The 4 cross-sectional surveys indicated significant reductions in parasite burden among the study population. The overall geometric mean parasite densities (GMPD) during first, second, third and fourth survey were 2229 infected red blood cells (irb)/µl, 353 irb/µl, 578 irb/µl and 184 irb/µl, respectively (ANOVA, F = 28.95, df = 3, 243, p < 0.0001) (Table 1). Geometric mean parasite density varied significantly among age groups during the first (ANOVA, F = 4.142, df = 2, 110, p = 0.018) and the third survey (ANOVA, F = 5.463, df = 2, 21, p = 0.012 (Fig. 3B). During the first and third surveys, children aged below 5 years old, had highest mean parasite densities compared to other age groups. However, during the second and fourth surveys, geometric mean parasite density did not vary significantly among age groups (ANOVA, F = 1.361, df = 2, 96, p = 0.26) and (ANOVA, F = 0.67, df = 2, 8, p = 0.54), respectively. The parasite density during each survey was categorized into low, moderate, high and hyperparasitaemia. The findings indicated elimination of high and hyperparasitaemia during the 4 cross-sectional surveys. For example, the study reported zero prevalence rates of hyperparasitaemia during the second, third and the fourth survey. Equally, during survey 3 and 4, there were no cases of high parasite density. With the onset of indoor residual spray program, most infections were due to low parasite densities as illustrated in Table 1.

### *Plasmodium* species distribution and *P. falciparum* gametocyte prevalence

Three malaria species were found in the study population with *P. falciparum* being most prevalent, followed by *P. malariae* and *P. ovale* as the least prevalent, as shown in Table 1. Both *P. malariae* and *P. ovale* occurred at low parasite densities hence mostly detected by PCR. Considering the prevalence of mono-infections during the 4 cross-sectional surveys, *Plasmodium falciparum* accounted for 94.3% (215/228), 81.6% (151/185), 68% (51/75) and 87.7% (50/57) of all infections during the first, second, third and fourth survey respectively. Similarly, the proportion of *P. malariae* during survey 1, 2, 3 and 4 was 0.9% (2/228), 8.6% (16/185), 10.7% (8/75) and 1.8% (1/57) respectively. The infections due to *P. ovale* only occurred during the first (0.4% (1/228), second (1.6% (3/185), and fourth survey (5.3% (3/57). Mixed species infections due to *P. falciparum* and *P. malariae* mostly occurred throughout the study period. Mixed infections involving *P. ovale* were rare and were reported during the first and second surveys, as illustrated in Table 2 below. Results further indicated lowest levels of gametocyte prevalence during survey periods. The declining trend of gametocyte prevalence was 1.3% (8/610), 0.5% (4/737), 0.14% (1/720) and 0.12% (1/849) during the first, second, third and the fourth survey respectively (*χ*^2^ = 12.97, df = 3, p = 0.005). The geometric mean gametocyte density was: 66.6, 35.9, 48 and 16 gametocytes/µl of blood during survey 1, 2, 3 and 4 respectively (ANOVA, F = 1.24, df = 3, 10, p = 0.35).

**Table 2:** *Plasmodium* species composition during the 4 cross-sectional survey as detected by qPCR

Survey period	*Plasmodium* species composition							
	*Pf*	*Pf* + *Pm*	*Pf* + *Pm* + *Po*	*Pf* + *Po*	*Pm*	*Pm* + *Po*	*Po*	Total
Feb 2018	215	7	2	1	2	0	1	228
Jun 2018	151	12	0	0	16	3	3	185
Feb 2019	52	15	0	0	8	0	0	75
Jun 2019	50	3	0	0	1	0	3	57

### Submicroscopic, asymptomatic and symptomatic *Plasmodium* infections

**Fig. 4:**
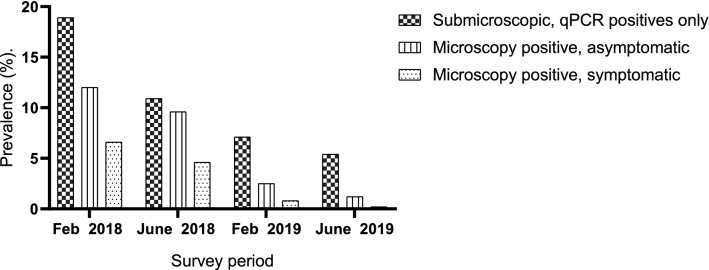
The graphs indicates the proportions of submicroscopic, asymptomatic and symptomatic infections during the four cross-sectional surveys

The absolute numbers of submicroscopic (qPCR positive alone) infections decreased during the four cross-sectional studies. For instance, the proportion of submicroscopic infections was 18.9% (115/610), 10.9% (80/737), 7.1% (51/720), and 5.4% (46/849) (*χ*^2^ = 80.2, df = 3, p < 0.0001) for the first, second, third and fourth surveys, respectively (Fig. 4). However, the relative proportion of submicroscopic infections increased during the four cross-sectional surveys. For example, the proportion of PCR positive infections that were submicroscopic increased from 50.4% (115/228) to 80.7% (46/57) over the study period (*χ*^2^ = 31.98, df = 3, p < 0.0001).

**Fig. 5:**
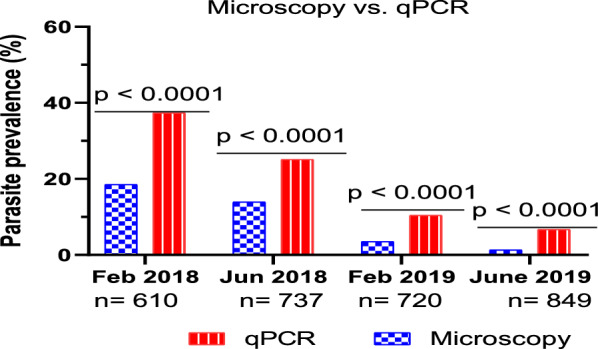
The graphs show the detection of malaria parasites by microscopy and qPCR during the 4 cross-sectional surveys

The rate of symptomatic infections as determined by microscopy decreased during the survey period. For instance, the rate of microscopy positive that were symptomatic during the first, second, third, and fourth surveys, was 6.6% (40/610), 4.6% (44/737), 0.8% (6/720), and 0.2% (2/849), respectively (*χ*^2^ = 78.6, df = 3, p < 0.0001) (Fig. 4). Similarly, the absolute numbers of microscopy positive that were asymptomatic during surveys 1,2,3 and 4, decreased from 12% (73/610) to 1.2% (9/849) (*χ*^2^ = 110.4, df = 3, p < 0.0001).. However, the relative proportions of microscopy positive that were asymptomatic increased from 64.6% (73/113) to 83.3% (9/11) (*χ*^2^ = 1.349, df = 3, p = 0.7). The detection of *Plasmodium* species infection rate by qPCR was significantly higher than that of microscopy as illustrated in Fig. 5.

## Discussion

This study was undertaken to document the impact of IRS and LLINs on parasitological profiles of malaria parasites in a human population in an endemic site in western Kenya. Accurate determination of *Plasmodium* infection rates is key towards monitoring the progress of malaria intervention programmes initiated within endemic regions [27]. To accurately document malaria prevalence rates within populations, more sensitive detection tools are essential. This will help detect infections including those with very low parasite densities which mostly go undetected by microscopy [28]. The present study investigated the prevalence rates of malaria parasites, asymptomatic and submicroscopic *Plasmodium* infections before the introduction of IRS and during two rounds of IRS in an area where LLINs were widely used. Four cross-sectional surveys were conducted and findings indicated a sustained reduction in *Plasmodium* infection rates especially after the rollout of IRS programme. Just before the onset of IRS, the *Plasmodium* prevalence rate by both microscopy and qPCR was at 18.5% and 37.4%, respectively. However, during the last survey, the prevalence of malaria parasites had declined to 1.3% by microscopy and 6.7% by qPCR.

The geometric mean parasite density significantly declined from 2229 parasites/µl of blood before intervention to 184 parasites/µl of blood after 2 rounds of IRS. The most plausible explanation to such a drastic reduction in parasite prevalence and parasite density could be due to very low parasite transmission after the application of IRS. For instance, in a neighboring Migori county, where a similar intervention was initiated, malaria transmission was reported to have declined significantly [29]. Furthermore, in Uganda [30], Mali [31] and Tanzania [32, 33] where IRS was initiated as a vector control strategy, the *Plasmodium* infection rate and parasite density decreased significantly.

Parasite prevalence determined by both microscopy and qPCR were reported to be highest among the age group 5–14 years old. This is in agreement with other previous studies [34–36]. A likely explanation to this trend has been highlighted in past studies [37–39]. For instance, majority in this age group are not under direct care of parents as compared to under 5 years old. They tend to be independent and most often may not sleep under bed nets or nets poorly placed, leaving them vulnerable to infectious mosquito bites. Poor health-seeking pattern has also been reported as a possible challenge in this age group, leading to more parasite infections [40], possibly because they have mild clinical symptoms. Unlike parasite prevalence, parasite density was highest in children under the age of 5, but decreased with age. Antiparasitic immunity develops with age, providing protection against high malaria parasite density [41, 42]. Due to repeated *Plasmodium* infections, older children, for example, develop an improved immune response that significantly suppresses the multiplication of asexual parasites.

The prevalence of asymptomatic and symptomatic cases decreased during the study period. This could be due to sustained malaria vector control in the study area through the use of IRS and LLINs. It was important to note, however, that the relative proportions of microscopy positives that were asymptomatic increased with each subsequent survey. This observed trend could be explained by a significant decrease in parasite prevalence and density. For example, the study revealed a complete elimination of hyperparasitaemia and high parasite density infections during the third and fourth survey. Likewise, there was a significant decrease in infections with moderate parasitaemia. As a result, the few existing *Plasmodium* infections with low parasite densities may be easily tolerated, increasing the number of asymptomatic cases. Similarly, the current study area has historically been a malaria endemic region. Areas undergoing perennial *Plasmodium* infections endure elevated rates of asymptomatic infections [43, 44]. This is due to increased tolerance towards parasite density [45]. Frequent exposure to *Plasmodium* infections may trigger strong immune modulation resulting in reduced clinical symptoms [46–49]. However, rising asymptomatic rates are likely to negate the gains and jeopardize vector control efforts in the study area.

The absolute number of submicroscopic infections decreased with decrease in *Plasmodium* infection rate. However, the relative proportion of submicroscopic infections increased during study period. As the parasite densities decrease, submicroscopic infections may increase due to microscopy’s low detection limit [12, 27]. This is comparable to previous studies, which reported high submicroscopic infections in low-transmission settings [50]. Submicroscopic infections are of public health interest as they pose serious health challenge to pregnant women [51] as well as causing mild anaemia [52]. The prevalence and density of gametocytes were extremely low and steadily declined with subsequent surveys. Although infectious gametocyte carriage is modulated by a variety of factors including immune response, treatment, and transmission intensity [53], the current study’s reduced levels of gametocytes could be attributed to a sustained vector control programme within the study area.

*Plasmodium* species detected in the current study varied with *P. falciparum* accounting for the highest proportions. This is consistent with other observations along the lake region, western Kenya [34, 54]. The study further reported existence of *P. malariae* and *P. ovale*. While the number of *P. falciparum* infection cases reduced, the proportion of *P. malariae* cases remained relatively stable over the course of four surveys. Most importantly, these *P. malariae* infections occurred at very minimal parasite densities, hence the majority of cases were diagnosed using qPCR. This is similar to a previous study which reported subpatent *P. malariae* with low parasite densities [55]. Low parasite density in *P. malariae* infections may be a challenge to microscopy based diagnosis leading to misdiagnosis or underreporting of malaria cases [55]. Accurate detection and treatment of *P. malariae* infections is paramount to minimize cases of nephrotic syndrome, which are associated with excessive mortality [56].

Detection rate of *Plasmodium* species by qPCR was significantly higher compared to that of microscopy. This is consistent with previous studies which compared the two diagnostic tools [57, 58]. Molecular based detection tools are known to be very sensitive hence able to identify cases with lowest parasite densities [59, 60]. Highly sensitive detection tools may be useful especially in settings where submicroscopic infections or co-infections with low parasite density *P. malariae* are common.

The study was limited to two sub-counties, hence the findings cannot not be generalized to the entire county. Besides, the study did not include the *Plasmodium* infection rate prior to net interventions. Finally, due to the study's cross-sectional design, it was difficult to directly link the lower *Plasmodium* infections to LLINs and IRS interventions.

## Conclusion

The study findings report significant reduction in *Plasmodium* infection rates and parasite densities following the introduction of the indoor residual spray programme. The reports also show that, while the absolute number of asymptomatic and submicroscopic infections decreased in the study area, the relative proportion of both asymptomatic and submicroscopic infections increased. This is likely to negate the progress achieved towards vector control. The study also demonstrated a large reduction in clinical malaria, which is an important public health benefit.

## Data Availability

The dataset used in this study is available from the corresponding author upon reasonable request.

## Abbreviations

LLINs: Long-lasting insecticidal nets
IRS: Indoor residual spray
DNA: Deoxyribonucleic acid
qPCR: Qualitative polymerase chain reaction
ICEMR: International center of excellence for malaria research
MoH: Ministry of Health
